# Effect of different concentrations of sufentanil combined with ropivacaine on epidural fever during labor: A single-center retrospective study

**DOI:** 10.1097/MD.0000000000038363

**Published:** 2024-05-31

**Authors:** Sujing Zhang, Yi You, Yu Huang, Chuantao Lin, Zhoujin Lin, Xiaoli Xue, Xiang Gao

**Affiliations:** a Department of Anesthesiology, Fujian Provincial Maternity and Child Health Hospital, Fuzhou, China; b College of Clinical Medicine for Obstetrics & Gynecology and Pediatrics, Fujian Medical University, Fuzhou, China.

**Keywords:** fever, pregnancy, ropivacaine, sufentanil

## Abstract

Labor epidural analgesia (LEA) is associated with increased maternal body temperature; however, the responsible mechanism is unknown. Recent studies suggest that changes in EA affect the incidence of fever and that epidural sufentanil supplementation enhances analgesia and reduces the amount of local anesthetic. The aim of this study was to evaluate the effect of different concentrations of sufentanil combined with ropivacaine on intrapartum fever during delivery. We performed a retrospective study comparing maternal fever rates in patients receiving labor analgesia between December 2018 and January 2019. Each patient receiving different concentrations of sufentanil in their EA received either proposal H (0.08% ropivacaine + 0.4 µg/mL sufentanil) or proposal L (0.08% ropivacaine + 0.2 µg/mL sufentanil), with the same nulliparous status. The primary outcome of this study was the incidence of intrapartum maternal fever, which was defined as any temperature ≥ 38°C during labor using Fisher exact test. Secondary outcome measures included visual analog scale (VAS) pain scores, birth events, and neonatal outcomes. We observed a perinatal fever incidence rate of 11.7% in the group receiving proposal L, while the incidence rate was 19.8% in the group receiving proposal H (*P* = .001). Five hours after administration, the average body temperature of the puerpera decreased significantly in the proposal L group compared with proposal H group. In addition, treatment with 0.2 µg/mL sufentanil provided satisfactory pain relief during labor, shortened the first stage of labor and total labor time, reduced oxytocin use, and had no significant adverse effects on neonatal outcomes. EA may increase the risk of intrapartum epidural-associated fever. Compared with the 0.4 µg/mL sufentanil group, the 0.2 µg/mL sufentanil group can provide better analgesia and improve maternal fever. These retrospective results highlighted the importance of prospective and mechanistic studies of maternal fever associated with intraspinal analgesia.

## 1. Introduction

Epidural analgesia (EA) is widely used to relieve pain during childbirth. Recently, there has been an increasing amount of evidence indicating that epidural labor analgesia is associated with an increase in maternal body temperature.^[1]^ This phenomenon was well described in comparison to the hypothermia associated with intraspinal block for cesarean section or non-obstetric procedures.^[2–4]^ However, the mechanism of the association between epidural labor analgesia and maternal hyperthermia remains unclear. It is likely to be the result of noninfectious inflammation caused by maternal immune activation, which is characterized as increasing levels of proinflammatory cytokines in the parturient.^[5,6]^ EA alters maternal thermoregulatory pathways, which may result in an inability to dissipate excessive heat, either as a direct result of sympathetic blockade^[7,8]^ or indirectly through reduced heat-dissipating activity in the absence of effective labor.^[9]^ Elevated maternal body temperature may lead not only to increased maternal heart rate, cardiac output, oxygen consumption, and catecholamine production, but also to increased fetal body temperature, the need for supplemental oxygen and mask ventilation, decreased neonatal birth rates, muscle tone and Apgar scores, and neonatal brain injury.^[10]^

Ropivacaine, a new long-acting amide local anesthetic, has the characteristics of low toxicity to cardiovascular and central nervous system. When used in low concentrations, it also has obvious characteristics of sensorimotor nerve separation; thus, it is specially used for labor analgesia and has no effect on the puerpera.^[11]^ Sufentanil is a selective μ receptor agonist with a rapid onset of action, shorter duration of action, stronger analgesic effect, and lower incidence of respiratory depression than morphine, alfentanil, and fentanyl. It is an ideal choice for EA.^[12]^ EA with ropivacaine and sufentanil has been used to promote painless labor and has been shown to be safe and effective.^[13,14]^

To the best of our knowledge, the effect of different dose combinations of sufentanil for EA on maternal body temperature has not been previously reported. Our single-center retrospective study of the effects of different doses of sufentanil in combination with ropivacaine for labor epidural analgesia (LEA) on primipara temperature, visual analog scale (VAS) pain scores, labor events, and neonatal outcomes during labor aims to further observe the mechanisms by which maternal fever is associated with epidural labor analgesia.

## 2. Methods

The Fujian Maternity and Child Health Hospital Review Committee approved this study **(2023KY053**) and waived the requirement for informed consent. We used an institutional obstetric anesthesia quality database to identify patients who received EA during labor between December 2018 and January 2019. We excluded patients who did not have a temperature measurement between the start of anesthesia care and delivery, multiparous women, gestational age ≤ 37 weeks, stillbirths, multiple pregnancies, metabolic diseases, such as diabetes, and high-risk pregnancy, preoperative fever, and admission to hospital due to intrauterine fetal death. From the electronic medical record, we collected data of 1203 women who underwent EA, 929 of whom met the inclusion criteria for gestational age. We recorded routine demographic and morphologic characteristics at delivery and admission, such as maternal age, height, white blood cell count, neutrophil count, VAS score before analgesia, BMI, and gestational age. Maternal vital signs including heart rate, arterial oxygen saturation and noninvasive blood pressure, uterine contractions and fetal heart rate were monitored as part of routine obstetric management and not as part of the study protocol per se; additionally, VAS scores were assessed. The VAS scores ranged from 0 to 10 cm, with the degree of pain increasing with increasing score. Induction of labor versus spontaneous delivery, mode of delivery, admission to hospital for delivery, status of rupture of membranes (ROM), time of delivery, oxytocin use, cervical dilation prior to epidural insertion, duration of analgesia, and other routine obstetric variables were recorded. After delivery, neonatal weight was recorded, as well as Apgar scores at 1 and 5 minutes and neonatal body temperature at birth and 12 hours after birth. All patients underwent epidural puncture at L2-3 or L3-4 during the study. Once the needle was in the epidural space, a 4 cm epidural catheter was placed on the cephalad and secured. Three milliliters of 1.5% lidocaine was injected and observed for 5 minutes to confirm that the mother had no subarachnoid block and local anesthetic toxicity. Two groups were used to initiate programmed intermittent epidural bolus (PIEB) or patient-controlled epidural analgesia (PCEA) for labor analgesia: high concentration sufentanil group (group H) with 0.4 µg/mL sufentanil + 0.08% ropivacaine; low concentration sufentanil group (group L) with 0.2 µg/mL sufentanil + 0.08% ropivacaine.

PIEB settings included an 8 mL bolus every 60 minutes; PCEA (demand) dose, 8 mL; maximum dose, 30 mL/h and lockout interval of 15 minutes. The parturients were instructed to press the PCEA device button if contractions were uncomfortable. We withdrew the epidural catheter after the end of the third stage of labor and terminated the analgesia protocol. Our delivery protocol requires that the temperature of the parturient be measured every hour after delivery analgesia. The parturient body temperature during the study was typically measured using a non-contact infrared thermometer (Braun pro4000). When the tympanic temperature is between 38°C and 38.5°C, we encourage the patient to drink more water and undergo physical cooling.

### 2.1. Statistical analysis

**After collecting cases for 2 months, we made preliminary statistics that the fever rate of group L was about 10%, and that of group H was 20%, power = 0.9, Alpha = 0.1, N2/N1 = 2, and the sample number was 352. Since the data we collected was larger than the required sample size for calculation, we did not continue to collect cases.**

Statistical analysis was performed using SAS software (SAS v9.4, SAS Institute, Cary, NC). The primary outcome was the incidence of maternal fever during delivery, which was defined as any body temperature ≧ 38°C before delivery and the change in maternal body temperature after analgesia. The rate of maternal fever in women with low and high concentrations of sufentanil was compared using Fisher exact test, reported as the percentage of women with fever in each group, and the difference between these 2 groups. The measurement data conforming to the normal distribution were recorded as mean ± SD, and the measurement data conforming to the skewed distribution were recorded as median and quadrilateral spacing. Analysis of variance was used to compare the data of 2 samples that fit the normal distribution and homogeneity of variance; the Mann–Whitney U test was used to compare the data of 2 samples that did not meet the above conditions; χ^2^ test was used for comparison of count data. A *P* value < .05 was considered significantly significant.

## 3. Result

**Figure 1.**
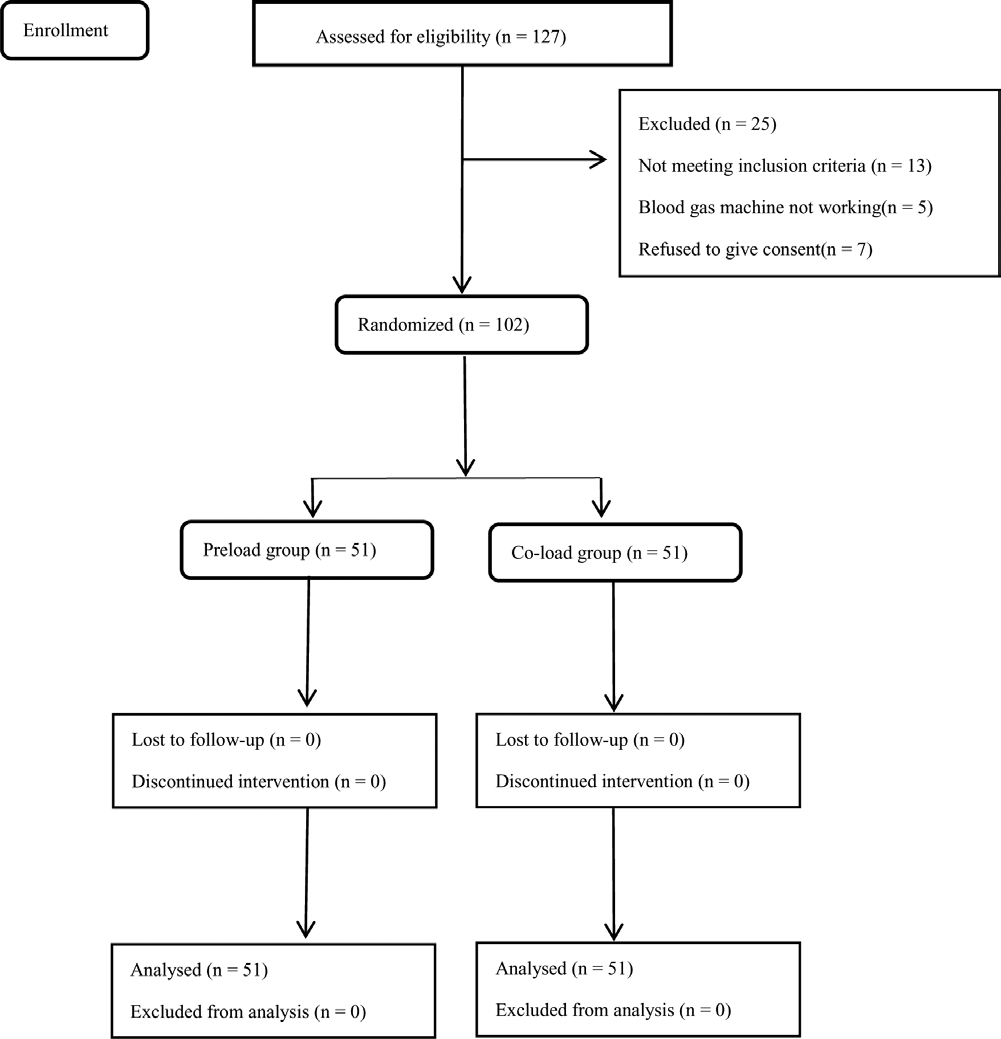
Study flow chart.

Our obstetric quality database included 1203 labor analgesia records during the study period. After excluding multiple pregnancies, stillbirth or twin pregnancy, and delivery of ≤ 37 weeks of gestation, intrauterine fetal death, and patients with multiple catheter types (e.g., epidural catheters replaced by spinal catheters), we included 587 women in group H (0.08% ropivacaine + 0.4 µg/mL sufentanil) and 342 women in group L in our study cohort (0.08% ropivacaine + 0.2 µg/mL sufentanil) (Fig. 1).

Maternal demographic characteristics, baseline cervical dilatation, and baseline body temperature were not significantly different between the 2 study groups (Table 1).

**Table 1:** Patient demographics.

	Group H (n = 587)	Group L (n = 342)	*P* value
Age	28.3 ± 3.350	28.0 ± 3.652	.164
BMI (kg/m^2^)	26.63 ± 9.254	26.07 ± 2.656	.223
Gestational age (wk)	38.26 ± 2.44	38.47 ± 2.71	.423
Baseline cervical dilatation (cm)	2.72 ± 1.33	2.87 ± 1.09	.522
White blood cell count (× 109/L)	9.63 ± 3.45784	9.62 ± 2.30113	.313
Neutrophils (× 109/L)	74.92 ± 27.2008	73.89 ± 7.1640	.355
VAS before analgesia (mm)	8.1243 ± 1.332	8.297 ± 1.16	.743

Data are expressed as n (%) or median (interquartile range). Comparisons between groups were performed using the Kruskal − Wallis test.

BMI = Body Mass Index, VAS = visual analog scale.

Most parturients (748/929, 80.52%) completed delivery within 8 hours after the start of analgesia. We analyzed the temperature within 8 hours and when the fetus was delivered.

### 3.1. Changes in maternal body temperature at delivery

**Figure 2.**
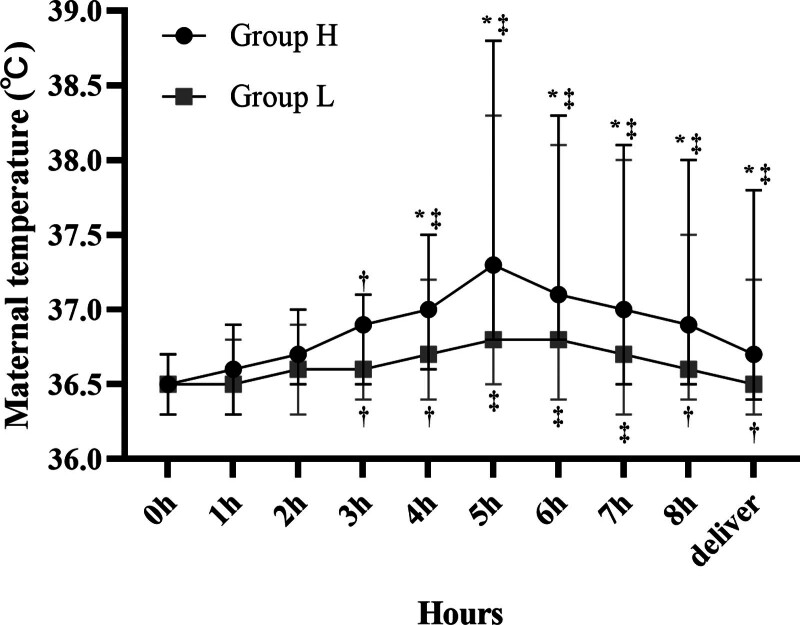
Average maternal body temperature at delivery. Maternal mean body temperature was lower in the 0.2 µg/mL sufentanil group than in the 0.4 µg/mL sufentanil group. [*] *P* < .05 vs group H, [†] *P* < .05 vs baseline, [‡] *P* < .01 vs baseline.

Maternal fever was less frequent in group L than in group H, with an incidence rate of 11.7% (45 of 342 patients) and 19.8% (116 of 587 patients), respectively (*P* = .001) (Table 2). Maternal temperature increased over time in both groups during labor (Fig. 2). In addition, the mean maternal body temperature increased significantly (*P* < .01) 5 hours after analgesia compared with that before EA in both groups. The mean maternal body temperature in group L was lower than that in group H 3 hours after analgesia. In addition, the primary outcome in Figure 1 was intrapartum maternal fever, defined as a body temperature ≥ 38°C before delivery.

**Table 2:** Birth events and neonatal outcomes in the 2 study groups.

Items	Group H (n = 587)	Group L (n = 342)	*P* value
Intrapartum fever (n (%))	116 (19.8)	40 (11.7)	.001
Duration of 1st stage (min)	649.±285	598 ± 237	.003
Duration of 2nd stage (min)	49 ± 28	46 ± 25	.103
Duration of 3rd stage (min)	6.5 ± 5.2	7.1 ± 6.8	.131
Total labor time (min)	703.7 ± 315.6	648.2 ± 259.7	.006
Duration of epidural (min)	575.3 ± 312.7	522.5 ± 257.9	.008
Bleeding volume at 2h postpartum (mL)	268.3 ± 47.5	271.8 ± 73.2	.377
Oxytocin augmentation (n (%))	111 (18.23)	45 (13.16)	.045
Instrument delivery (n (%))	47 (8.01)	24 (7.02)	.611
Cesarean delivery (n (%))	123 (20.95)	85 (24.85)	.192
ROM (%)	200 (34.07)	116 (33.92)	.887
Urinary retention (n (%))	121 (20.61)	55 (16.08)	.099
Birth weight (g)	3201.25 ± 326.96	3165.81 ± 558.31	.443
Apgar score at 1 min	9.86 ± 0.43	9.79 ± 0.46	.607
Apgar score at 5 min	10.00 ± 0.00	9.97 ± 0.53	.819
Body temperature at birth at 1 min	36.01 ± 0.932	36.11 ± 0.711	.739
Body temperature at birth at 12 h	36.43 ± 0.277	36.55 ± 0.384	.933

ROM: status of rupture of membranes; Data are expressed as n (%) or median (interquartile range); comparisons between groups were performed using Fisher exact or Kruskal − Wallis test.

### 3.2. VAS pain score during delivery

**Figure 3.**
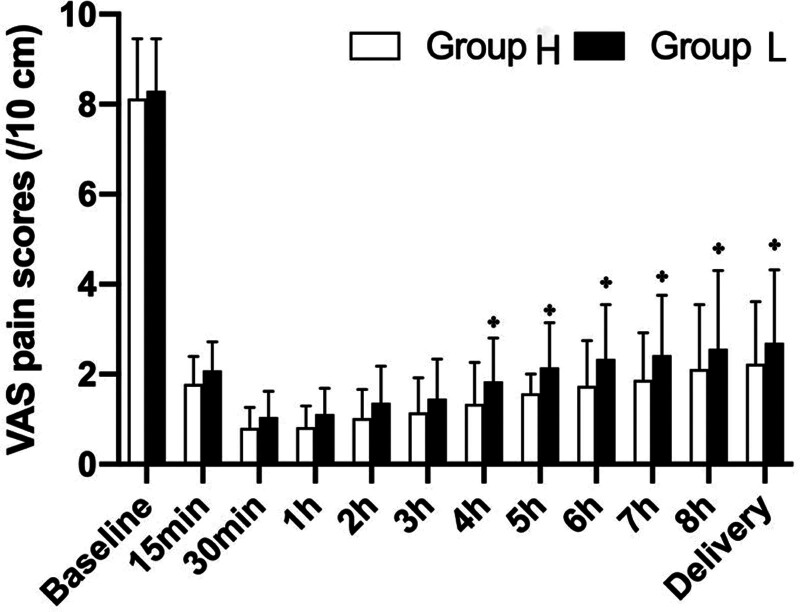
Visual analog scale (VAS) pain scores during labor. [*] *P* < .05 vs Group H.

As shown in Figure 3, the mean VAS pain scores of the 2 groups showed similar changes in the present study. Specifically, in the early stage of labor analgesia, it gradually decreased over time to <3 cm from 15 minutes after the onset of EA, indicating effective pain relief in both groups. While group L had significantly higher VAS pain scores than group H at the study time point after analgesia, satisfactory pain relief was still observed (<3 cm).

### 3.3. Delivery events and neonatal outcomes

Birth events and neonatal outcomes were analyzed in relation to the type of analgesics used, and the results are presented in Table 2. Compared with those in group H, the duration of first stage, total labor time, EA, and the dosage of oxytocin were decreased in group L (*P* < .05). There was no significant difference in other delivery events such as mode of delivery and urinary retention between the 2 groups. Neonatal outcomes, Apgar scores at 1 and 5 mins, and postnatal neonatal temperature were also similar between the groups.

## 4. Discussion

LEA is currently recognized as the most widely used analgesic method with the best analgesic effect^[15]^; however, it does have some problems. Among them, intrapartum fever is the focus of current research.^[16]^ Intrapartum fever may result from infection, such as chorioamnionitis, or from noninfectious factors, such as LEA. Pregnant women with intrapartum fever have a risk of antibiotic treatment and surgical delivery, while their newborns may be admitted to the neonatal intensive care unit.^[2,17]^ In this study, our data suggests that maternal body temperature tends to increase gradually during labor in both treatment groups, which was consistent with previous studies^[18]^ and that the 0.2 µg/mL sufentanil group had a lower incidence of maternal fever than the 0.4 µg/mL sufentanil group and provided satisfactory pain relief during labor. In addition, EA received by group L shortened the first stage of labor, total labor time, and reducing the use of oxytocin had no significant adverse effects on neonatal outcomes.

At present, the commonly used EA are ropivacaine combined with sufentanil. Ropivacaine is a new amide long-acting local anesthetic, whose chemical structure is similar to that of bupivacaine and mepivacaine, although the nitrogen side chain of n-hexane is replaced by propyl.^[19]^ The addition of ropivacaine increases the incidence of fever during labor.^[20]^ Sufentanil enhances analgesia and reduces ropivacaine consumption.^[11]^ The mechanism of the effect of opioids on body temperature is unknown, as different opioid drugs act on different opioid receptors located within the preoptic area (POA) of the anterior hypothalamus and play different roles in thermoregulation.^[16,21]^ Sufentanil is approximately 1000 times more potent than morphine^[20]^ and has a selective bond and high affinity for the μ1 site. It has been reported that μ receptors have an effect on the development of hyperthermia.^[16,22]^ Epidural sufentanil can be absorbed into the blood or penetrate the cerebrospinal fluid, and the afferent pathway starts from primary thermal sensory neurons, which produce different changes in temperature sensation through the spino-thalamo-cortical pathway.^[23]^ Changes in spinal cord temperature can affect the activity of thermoregulatory neurons in the POA,^[24]^ and previous studies have shown that systemic administration of opioids mainly produces a biphasic effect on the body temperature of mice, as follows: low-dose hyperthermia and high-dose hypothermia.^[25]^ Our study results that epidural administration of a low dose of sufentanil induces intrapartum fever further confirmed the results of previous studies.

It is well established that thermoregulatory factors are involved in the incidence of EA-associated fever. Under physiological circumstances, the balance between heat production and heat dissipation kept body temperature at a normal level. In response to labor, the laboring woman metabolic demand and minute ventilation increase during contractions.^[18]^ Maternal hyperventilation and perspiration can dissipate the increased heat energy due to increased metabolic consumption. The disturbance of thermogenesis and heat dissipation mechanism caused by EA may lead to body temperature retention, which can cause maternal hyperthermia. Labor analgesia blocks the sympathetic nerve of the lower limbs of the puerpera, inhibits the perspiration system of the puerpera, dilates the regional blood vessels, slows down the heat loss, and leads to heat accumulation. With the extension of labor analgesia time, the body temperature of the puerpera will gradually rise and reach the peak value after 5 hours.^[26]^ In addition, prolonged labor is a risk factor for EA and fever.^[27]^ A recent study^[28]^ showed that duration of total EA >6.3 hours was associated with increased duration of maternal fever during labor and early measures, such as the use of oxytocin to accelerate labor, which is similar to our findings. Several clinical studies have found that the use of opioid analgesics can prolong labor time,^[26,27]^ which may be partly due to the inhibitory effect of opioids on the uterine contractile force. A study on the effects of opioids on the uterine muscles of an isolated human pregnancy showed that fentanyl and meperidine inhibited uterine contractility in a concentration-dependent manner and pretreatment with either naloxone, N(G)-nitro-L-arginine methyl ester, atenolol, or indomethacin did not affect the uterine responses to opioids.^[29]^

These findings suggest that the duration of EA is important for the occurrence of maternal fever. In this study, we found that the total duration of labor was shorter in group L than in group H, and we found that the VAS pain score was higher in the 0.2 µg/mL group than in the 0.4 µg/mL ropivacaine group, indicating that labor pain relief was less, but still satisfactory in the L group (<3 cm). There was substantial evidence that painful stimulation during labor can cause hyperventilation, which contributes to heat loss in laboring women.^[14]^ Thus, our data suggests that the effect of 0.2 µg/mL sufentanil on fever associated with EA may be due to the shortening of labor, while it partially restores the disturbance of the thermogenic–dissipating mechanism caused by EA.

Our study found that the oxytocin used by 0.2 µg/mL sufentanil in nulliparous women during labor was significantly less than that of 0.4 µg/mL sufentanil. The link between increased oxytocin and labor fever has been extensively studied in previous studies. Numerous studies have shown that the cause of oxytocin-induced intrapartum fever may be that oxytocin promotes the release of inflammatory mediator F and positive regulatory mediator prostaglandin E2, resulting in an upward shift of the set point of the thermoregulatory center.^[29]^ Therefore, we believe that the reduction in oxytocin administration caused by 0.2 µg/mL sufentanil treatment may reduce the secretion of inflammatory mediators, which may also contribute to its beneficial effect on fever associated with EA.

Postnatal neonatal status was also an important outcome in our study. Apgar scores ranged from 9 to 10 for sufentanil concentrations in both groups, and no infant had a temperature above 38°C, with no significant difference between the 2 groups. This suggests that epidural labor analgesia has little effect on the newborn. In our study, there were no significant differences in white blood cell counts, neutrophil counts, or maternal baseline body temperature between the 2 groups before the start of analgesia.

As the data were mainly recorded by electronic medical record, there were some limitations in our study, as follows: The parturients were not randomized, and the analgesia proposals they received were administered by different anesthesiologists, possibly with personal bias. As most of the women in this study were in labor for <8 hours, we only tracked maternal body temperature for 8 hours after the starting of delivery; hence, we may have missed any differences that may have occurred after that time period. 3. The sample size was small and included only a small number of febrile women; therefore, we could not strictly control all potential confounders. 4. Epidural administration of small doses of sufentanil is safe, but there is still a potential risk of side effects, such as nausea, vomiting, urinary retention, and itching. We did not follow women with these side effects to analyze long-term maternal effects.

## 5. Conclusion

In conclusion, this retrospective study demonstrated that the use of intermittent epidural labor analgesia with 0.2 µg/mL sufentanil + 0.08% ropivacaine alleviated maternal fever during labor as well shortened the first stage and total labor time. The partial restoration of the disturbance of the thermogenic–dissipating mechanism and the reduction of oxytocin use may together lead to this beneficial effect. In addition, treatment with 0.2 µg/mL sufentanil continued to provide satisfactory pain relief during labor. Our results suggest that 0.2 µg/mL sufentanil + 0.08% ropivacaine for EA may be a good option for EA during labor.

## Author contributions

**Conceptualization:** Yu Huang, Chuantao Lin.

**Data curation:** Yi You, Xiaoli Xue.

**Formal analysis:** Yi You.

**Investigation:** Sujing Zhang, Yu Huang.

**Methodology:** Sujing Zhang, Xiang Gao.

**Supervision:** Xiang Gao.

**Validation:** Zhoujin Lin, Xiaoli Xue.

**Writing – original draft:** Sujing Zhang, Yi You.
